# Three-dimensional surgical simulation improves the planning for correction of facial prognathism and asymmetry: A qualitative and quantitative study

**DOI:** 10.1038/srep40423

**Published:** 2017-01-10

**Authors:** Cheng-Ting Ho, Hsiu-Hsia Lin, Eric J. W. Liou, Lun-Jou Lo

**Affiliations:** 1 Department of Craniofacial Orthodontics, Department of Dentistry, Chang Gung Memorial Hospital, Taoyuan, Taiwan; 2 Department of Craniofacial Research Center, Chang Gung Memorial Hospital, Taoyuan, Taiwan; 3 Department of Plastic & Reconstructive Surgery, and Craniofacial Research Center, Chang Gung Memorial Hospital, Chang Gung University, Taoyuan, Taiwan

## Abstract

Traditional planning method for orthognathic surgery has limitations of cephalometric analysis, especially for patients with asymmetry. The aim of this study was to assess surgical plan modification after 3-demensional (3D) simulation. The procedures were to perform traditional surgical planning, construction of 3D model for the initial surgical plan (P1), 3D model of altered surgical plan after simulation (P2), comparison between P1 and P2 models, surgical execution, and postoperative validation using superimposition and root-mean-square difference (RMSD) between postoperative 3D image and P2 simulation model. Surgical plan was modified after 3D simulation in 93% of the cases. Absolute linear changes of landmarks in mediolateral direction (x-axis) were significant and between 1.11 to 1.62 mm. The pitch, yaw, and roll rotation as well as ramus inclination correction also showed significant changes after the 3D planning. Yaw rotation of the maxillomandibular complex (1.88 ± 0.32°) and change of ramus inclination (3.37 ± 3.21°) were most frequently performed for correction of the facial asymmetry. Errors between the postsurgical image and 3D simulation were acceptable, with RMSD 0.63 ± 0.25 mm for the maxilla and 0.85 ± 0.41 mm for the mandible. The information from this study could be used to augment the clinical planning and surgical execution when a conventional approach is applied.

Two-dimensional (2D) radiography and model surgery have traditionally been used for planning orthognathic surgeries. This approach, however, has limitations when presenting and analyzing complex three-dimensional (3D) maxillofacial structures, especially for patients with major facial deformity or asymmetry^1^,^2^,^3^. Because 2D cephalometric images cannot provide complete information about the 3D structures, when conventional 2D surgical plans are executed, unexpected problems–such as bony collision in the ramus area; discrepancy in pitch, roll, and yaw rotation; midline difference; and chin inadequacy–may occur^4^,^5^,^6^,^7^, leading to unsatisfactory outcomes.

Three-dimensional imaging has revolutionized orthodontic and orthognathic surgical planning^8^,^9^,^10^. For patients with facial asymmetry, a 3D model can demonstrate the extent of yaw rotation in the maxilla and mandible, occlusal plane canting, and differential length on a mandibular body or the ramus. Compared with 2D images, 3D images register more accurate anatomic information and enable more precise quantitative measurement^11^,^12^,^13^. Moreover, 3D images are more effective in diagnosing asymmetry and estimating severity and thus may necessitate changes in surgical designs based on traditional 2D modelling^14^.

Low-dose cone-beam computed tomography (CBCT), which enables the accurate reconstruction of a 3D skeletal model, has been applied widely in treatment planning for orthognathic surgeries^15^,^16^,^17^,^18^. Computer-aided design and manufacturing (CAD/CAM) techniques have been adopted to enhance the accuracy of preoperative planning and guidance for surgical procedures^19^,^20^,^21^,^22^,^23^,^24^. 3D virtual surgery was started and applied in orthognathic surgery for correction of facial deformities^25^,^26^,^27^,^28^. Recently developed 3D computer-assisted orthognathic surgery systems incorporate advanced 3D imaging, computer simulation software, CAD/CAM techniques, and image-guidance technologies and offer the high level of precision essential for optimal treatment planning and intraoperative execution^29^,^30^,^31^. Xia *et al*. and Gateno *et al*. have developed a computer-aided surgical simulation system that can create a composite skull model and simulate and transfer a virtual plan for correcting complex craniomaxillofacial deformities^32^,^33^,^34^. Similarly, Bell *et al*. combined computer planning and intraoperative navigation^35^,^36^, and Lucia *et al*. presented a computer-aided surgery system that provides surgical planning and simulation and intraoperative guidance^37^. Lin *et al*. proposed a protocol for assessing the surgical simulation, guide positioning, intraoperative navigation, and outcome validation provided by computer-assisted surgery systems^38^,^39^. The 3D virtual surgery combined with navigation was proposed and suggested that simulation-guided navigation makes accurate postoperative outcomes possible for maxillary repositioning in orthognathic surgery^40^,^41^,^42^,^43^,^44^,^45^. However, the improvement from the 3D technique over the conventional method should be quantitated.

A 2D plan can be conveniently revised after 3D surgical simulation. Few studies have investigated the quantitative assessment of such revision in terms of midline correction; chin position; yaw, roll, and pitch rotation; and frontal ramus inclination. This study evaluated how conventional 2D surgical plans were modified after 3D computer-assisted surgical simulation for patients with class III malocclusion and facial asymmetry.

## Results

The ICCs ranged from 0.85 to 0.96 for measurements in final 3D planning model (P2) within and between observers, and ranged from 0.81 to 0.92 for displacements of the 23 landmark locations in comparison of the P1 and P2 models, indicating acceptable intraobserver reproducibility and interobserver reliability. Absolute linear differences in the landmarks of the (x, y, z) coordinates of the P1 (2D) and P2 (3D) models are listed in Table 1; landmarks with a difference of more than 0.5 mm were considered to have changed. All absolute linear differences along the mediolateral direction (x axis) were significant and were more than 1 mm, indicating midline correction. The lower molars L6L and L6R had the largest change of 1.7 mm. Absolute linear differences along the inferosuperior direction (y axis) were not as large as those along the x axis. Significant vertical changes (i.e., canting correction) were observed at the U3, U6R, U6L, L6R, L6L, and chin point. Absolute linear differences of landmarks along the anteroposterior direction (z axis) were smaller than those along the x and y axes; significant changes in the anteroposterior direction were observed only at B, U6L, and U6R.

Absolute angular differences of planes in the 2D and 3D plans are detailed in Table 2. Significant changes were noted in the pitch, yaw, and roll rotation as well as ramus inclination correction. Frontal ramus inclination had the largest correction of 3.37°, followed by yaw (1.88°), pitch (1.73°), and roll (1.06°).

**Figure 1:**
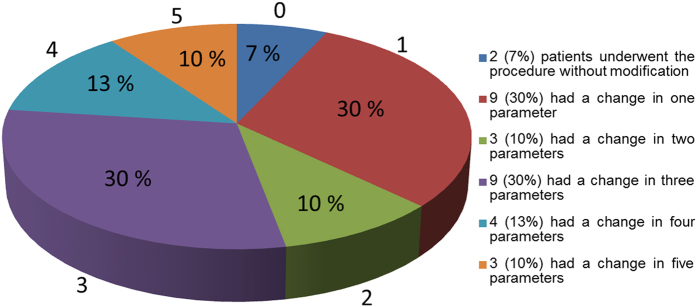
The Pie chart showing the percentage of patients with different numbers of parameter change.

**Figure 2:**
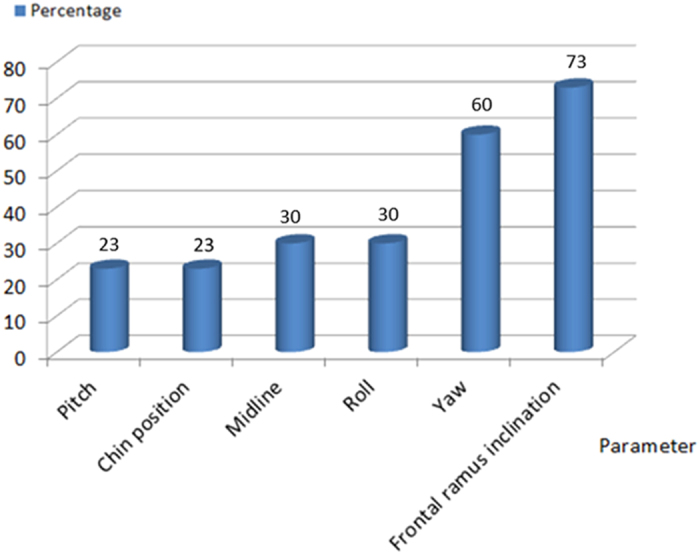
The frequency of parameter changes in the patients.

In the 30 patients in this study, the distribution and percentage of MMC positional changes from the 2D to the 3D plan were evaluated in terms of 6 parameters: midline, yaw, roll, pitch, ramus inclination, and chin position. Only for 2 patients (7%) were no changes necessary in the 2D plan. For obtaining the best outcomes, for 9 patients (30%), changes were necessary in 1 parameter; similarly, for 3 (10%), 9 (30%), 4 (13%), and 3 (10%) patients, changes were necessary in 2, 3, 4, and 5 parameters, respectively (Fig. 1). Thus, 93% of the patients in this study required changes in at least 1 and up to 5 parameters in their 2D plan; the most frequently changed parameters were frontal ramus inclination (73%) and yaw rotation (60%), followed by roll (30%), midline correction (30%), pitch rotation (23%), and chin position correction (23%) (Fig. 2).

The accuracy of the surgical outcome and 3D simulation were evaluated by calculating the root-mean-square difference (RMSD) of the 3D simulation and postsurgical CBCT images (Table 3). The maxilla had a lower RMSD (0.63 mm) than did the mandible (0.85 mm). Overall, the accuracy of the surgical execution of the 3D plan was acceptable.

**Figure 3:**
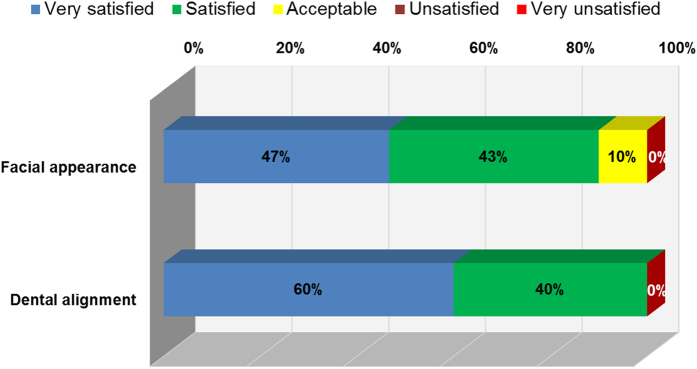
Outcome of patient satisfaction at least 6 months after the orthognathic surgery for correction of prognathism and facial asymmetry.

An investigation of patient satisfaction revealed that 90% of the patients were happy with the surgical outcome (i.e., facial appearance), and all patients were satisfied with the dental alignment (Fig. 3). No patients in this series were dissatisfied, and none received secondary correction for the initial facial deformity.

## Discussion

Advances in computer-aided surgical simulation has resulted in the wide use of new clinical protocols to evaluate craniomaxillofacial deformity and plan surgical procedures^4^, especially for orthognathic surgeries as these protocols enable planning through virtual osteotomy in patients’ 3D models. In addition, positioning guides and dental splints can be fabricated through computer-aided methods for the accurate fixation of the MMC to the cranium^6^. Xia *et al*. reported that the surgical outcomes achieved with the use of computer-aided surgical simulation were better than those achieved through traditional methods. Moreover, they reported that computer-aided techniques enable the surgeon to correct maxillary yaw deformities, place proximal/distal segments, and accurately restore mandibular symmetry. The present authors agree with their report, and the differences or the improvement were further quantitated. An increasing number of orthognathic surgeries are now being planned through computer-assisted methods. Virtual models detect and correct concealed problems and consequently assist in obtaining more favorable outcome.

The results of this study suggest that 3D planning outperforms 2D planning in correcting midline deviation, ramus asymmetry, occlusal plane canting, and chin position. Absolute linear differences of landmarks along the x axis were significant (1.11–1.62 mm, Table 1) and indicated that the midsagittal landmarks (A, U1, L1, B, Pog, and Me) moved toward the skeletal midline in the 3D plan. The molars required the largest number of changes in the left–right direction, 1.62 mm for maxillary molars and 1.52 mm for mandibular molars, possibly because of their longer radius from the rotation center. The absolute linear differences of landmarks along the y axis ranged from 0.50 to 1.17 mm and were less than those along the x axis, with only 6 landmarks moving significantly. The significant changes of landmarks U6R, U6L, L6R and L6L along the y axis indicated equivalent distance correction from the molars to the FH plane on both sides and more accurate occlusal cant correction. The absolute linear differences of landmarks along the z axis were 0.50 to 1.17 mm and were less significant, except for landmarks B, U6L, and U6R, revealing that the 3D plan did not differ much from the 2D plan along the anteroposterior direction. Generally, after 3D simulation, landmarks along the mandible were more displaced than those on the maxilla.

Midline correction varied from 1 to 2 mm (Table 1). Previous reports vary in their depiction of the magnitude of midline deviation without reducing smile aesthetics. Pinho *et al*. stated that 1 mm maximum acceptable deviation^46^. However, other studies have suggested that a midline deviation of up to 2 mm is acceptable^47^,^48^,^49^,^50^ and that medical personnel are more sensitive to midline discrepancy than are laypeople. The difference up to 1.6 mm between the two planning systems might not have obvious clinical impact. However, the 3D system is still more accurate than the conventional 2D method, and the extra accuracy could camouflage minor human error during the surgery. In addition to the midline problem, this study revealed that the 2D plan could not completely predict occlusal cant and yaw correction, which may compromise dentofacial aesthetics.

Angular changes in the MMC after 3D simulation were significant (Table 2). The yaw rotation was changed by 1.88° to obtain proper alignment of proximal/distal segments and more symmetry along the ramus. Yaw rotation is difficult to predict in 2D planning but is vital for managing intraoperative bony collision and cheek asymmetry. Frontal ramus inclination decreased from 4.14° to 0.78° resulting in more cheek symmetry after the simulation because the proximal segment can be moved in to or out from the inner cortex before the bicortical screw fixation. For patients complaining cheek asymmetry, the position of the ramus segments must be changed to achieve symmetry. Pitch rotation was changed by 1.73° as clockwise rotation of the MMC was planned to correct the concave facial profile with class III malocclusion, increasing postoperative stability and obtaining a better smile arc^51^. Figure 2 presents the distribution of the changes in the parameters; the frontal ramus inclination and yaw rotation were the most altered. During 3D virtual surgery, especially for patients with facial asymmetry, movement of the proximal segment of mandibular ramus is required in order to achieve symmetry on both sides. The authors simulated the axis or fulcrum of the ramus rotation using the condylar center, and degree of movement was decided by the symmetry from both ramus inclinations. We also used a CAD/CAM positioning (spacer) guides to facilitate reproduction of the planned ramus position, allowing precise proximal segment positioning. Yaw rotation of the MMC may change anteroposterior projection on both sides of the maxilla. In our experience, the discrepancy in the maxillary anteroposterior dimension is less noticeable, more tolerated, and easier to correct than the discrepancy in the ramus.

Roll, midline, chin position, and pitch were less frequently revised (Fig. 2) because these can be predicted on 2D images. However, the 20–30% incidence of change after simulation indicated that the 3D method is more sensitive and can further improve surgical planning. Occlusal canting on 2D posteroanterior radiograph may have been miscalculated because of the difficulty in precisely defining molar crown position, which can be readily identified on a 3D model. Therefore, canting correction (roll) was required for 30% of the patients.

For most patients with facial asymmetry, the original 2D surgical plan must be revised after computer-assisted simulation. In this study, 93% of the patients required revisions in 1–5 parameters. In our experience, severity of facial deformity is proportional to the extent of the change in the parameter: 10% of the patients required changes in 5 parameters because of severe canting and facial asymmetry. Thus, the traditional 2D method is inadequate in treating patients with facial asymmetry.

**Figure 4:**
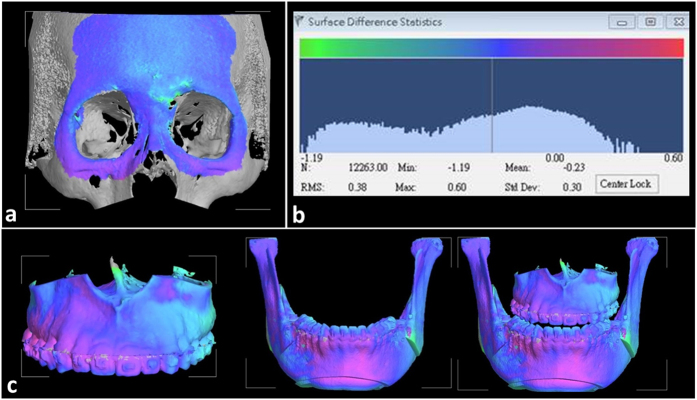
The discrepancy of superimposition of models was represented in the color-coded visualization and presented in terms of RMSD value (**a**). Initial registration of virtual planning and postoperative CBCT model (**b**). The RMSD distribution and statistics (**c**). The discrepancy of superimposition of virtual surgery and postsurgical images in maxilla, mandible and maxilla-mandibular complex segments. The blue color indicated the no deviation on both surfaces, other colors showed different degree of deviation.

This study focused on how conventional 2D surgical plan was revised after 3D computer-assisted surgical simulation for patients with asymmetric facial prognathism. It is to be noted that there is difference in the planning and outcome assessment between the two systems, and critical analysis of the difference is required. The study did not intend to overlook the well-established 2D conventional planning for orthognathic surgery, but rather to draw attentions to acknowledge the deficit of 2D method and the possible modifications into clinical planning and surgical execution, so that better outcome could be achieved. Considering the additional time, expense and effort required, the 3D computer-assisted surgical simulation system might be considered as an additional or alternative method for planning patients with complex maxillofacial deformity. As for the accuracy of the actual reproduction of the planning on the real patient, the positioning guide combined with the single-splint surgical technique used to control the movement of the MMC proposed in this study was effective and user friendly. The hooked shape design of the guide can be quickly and accurately wedged into the maxillary piriform edge, and the temporary screw fixation of the MMC repositioning guide facilitates intraoperative inspection. A facial aesthetic outcome is as important as functional dental results. A real-time navigation can be used as a definitive tool to determine the final bone position without the physical guides or as an additional tool to guide the bone movement. In this study, the accuracy of reproduction of the 3D plan was tested using the RMSD value between the 3D simulation image and the postoperative model. Accurate registration of the two image models is important before the comparisons. 3D simulation and postoperative models were superimposed using the surface registration method on the basis of the cranial base, which was stable and unaffected by the surgery. The accuracy of the cranial base registration was verified by observing the distance color map between two superimposed images. Based on the color scale, differences between the surfaces was less than 0.3 mm over the forehead and cranial base regions and the RMDS value of forehead and orbital areas were calculated and used to evaluate the front-orbital matching error (Fig. 4). The RMSD value of 0.5 mm or less was considered acceptable to ensure that the corresponding reference areas had the maximum possible accuracy^52^,^53^. Overall, the accuracy of the surgical execution achieved a satisfactory precision in positioning the maxilla with an acceptable RMSD of the maxilla (0.63 mm) and the mandible (0.85 mm) (Table 3).

In summary, the 3D computer assisted surgical simulation helps to improve the planning for patients complaining facial prognathism and asymmetry. The revisions of planning occurred more in the frontal ramus inclination and yaw rotation, but also in other movements of the maxillomandibular complex. The improvement of the surgical planning was measured. The treatment outcome was satisfactory. The information from this study could be used to augment the clinical planning and surgical execution when a conventional approach is applied.

## Methods

**Figure 5:**
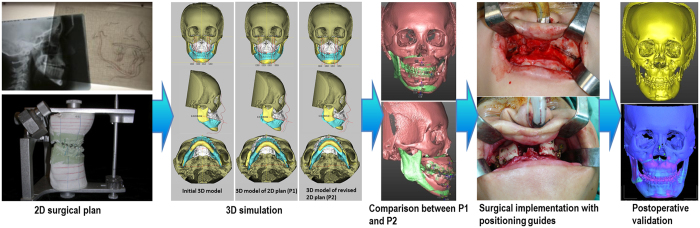
Flow chart of this study procedure.

The study procedure, outlined in Fig. 5, was divided into five stages: (I) traditional 2D surgical planning, (II) 3D simulation—3D CT images and initial models, 3D models of the 2D plans (P1), modified 3D models after simulation (P2), (III) comparison between P1 and P2, (IV) surgical execution aided by positioning guides, and (V) postoperative validation—superimposition of postoperative 3D images and the P2 models.

### Ethics

This retrospective study was conducted and approved by Chang Gung Craniofacial Center, Taiwan. All experiments were performed with the approval of the Institutional Review Board (IRB) of Chang Gung Memorial Hospital (IRB 103-4038C) and the study methods were carried out in accordance with the approved guidelines of IRB. Written informed consents were obtained from the patients or the guardians of the patients younger than 20 years.

### Patient collection

This study included thirty consecutive patients (22 female and 8 male) with class III malocclusion and facial asymmetry undergoing corrective surgery from July 2013 to Feb 2015. The mean age of these patients at surgery was 22.4 years (18–26 years). Patients with congenital or acquired deformities and syndromes, facial cleft, or a history of trauma were excluded. All patients had a concave facial profile, paranasal depression, mandibular prognathism, class III malocclusion, an inverse incisal relationship (overjet −2.5 ± 1.66 mm), and facial asymmetry. The facial asymmetry was characterized by occlusal plane canting, discrepancy between upper and lower dental midline, chin deviation away from the midline, and cheek and jaw irregularity that was readily perceivable by patients and clinicians. Psychosocial evaluation yielded negative findings in these patients.

### Image acquisition

Three-dimensional maxillofacial images were acquired using an i-CAT CBCT scanner (Imaging Sciences International, Hatfield, PA) with a low-dose protocol and patient teeth in light-contact condition at 120 kV, 5 mA, and 50 Hz. The extended field of view was 22 (height) × 16 (depth) cm, the scanning time was 40 s, and the voxel size was 0.4 × 0.4 × 0.4 mm. The images were stored in the digital imaging and communications in medicine (DICOM) format and processed with a slice thickness of 0.4 mm. Three-dimensional images and the initial 3D virtual models were obtained using CBCT and the SimPlant O&O (Materialize, Leuven, Belgium) and Dolphin (Dolphin Imaging and Management solutions, Chatsworth, California) software programs. Using the segmentation function, the maxilla and mandible were outlined and the replacement of dentition in 3D CBCT and the virtual occlusion setup were obtained.

### 2D surgical planning

All patients received presurgical orthodontic treatment, including leveling, alignment, arch coordination, and dental decompensation. One week before the surgery, 2D cephalogram (lateral and frontal views), photographs, and dental casts were captured and the CBCT examination was performed. Subsequently, 2D surgical planning was conducted according to the cephalometrics for the orthognathic surgery^51^,^54^. The dental casts were mounted in an articulator with facebow transfer and bite registration. The model surgery was performed according to the surgical plan drawn from the 2D cephalometrics, and the final occlusal splint was fabricated according to the planned maxilla and mandibular position. All aforementioned procedures were performed by the same orthodontist (CTH).

### 3D surgical simulation

The 2D surgical plan was transferred to the 3D simulation system, and an initial virtual surgical model was created. A Frankfort horizontal (FH) plane was used as the reference plane. A Frankfort horizontal (FH) plane was used as the reference plane. The FH plane was drawn from orbitale to average porion in 2D lateral cephalometric radiographs. The FH plane passed through the midpoint between left and right porion, right orbitale, and left orbitale in 3D model. Although the FH plane of a 2D planning could be somewhat different from the FH of a 3D environment. Comparing 2D and 3D system, some corresponding measurement such as A-B, ANS-Me, N-ANS, S-ANS, ANS-U1, SNA, SNB, MPA and upper occlusal plane to FH plane have found no clinically significant difference. These 2D measurements could be substituted by the corresponding 3D measurements on the 3D model as the registration of the landmarks were inspected in cross-sectional images of axial, coronal, and sagittal slices which 3D Frankfort horizontal (FH) plane was the most concordant with FH plane used for cephalometric radiography^11^,^52^,^53^. There were significant differences in gonial angle, lateral ramal inclinations and ramus length between 2D and 3D. As we know, a lateral cephalometric radiograph projects an object slanted toward the left or right onto a sagittal plane, an error in the length or angle depend on how much it slants. Thus, it is less effective in measuring the ramus. It also may be difficult to find asymmetry in case the maxilla yaw on the vertical axis of the skull and this can be solved by 3D measurement. The 3D surgical model included LeFort I osteotomy in the maxilla, bilateral sagittal split osteotomy in the mandibular ramus, and genioplasty. The single-splint two-jaw orthognathic surgery method was applied. The distal mandibular segment was moved by using the final occlusal splint such that it occluded the maxilla, thus forming the maxillomandibular complex (MMC). The MMC was mobilized to the planned position (labelled P1) according to the 2D surgical plan. Data used for transferring 2D plan to 3D system were (1) the midline shift and roll adjustment on the frontal view, (2) pitch rotation, positioning of A, ANS, U1, U6, SNA, and SNB in the right lateral view, and (3) genioplasty in both frontal and right lateral views. The MMC position was evaluated and modified if it was found not perfect. The improvement of the 2D planning in 3D environment was adjusted by using certain measurements that are much closure to Chinese norms of cephalometric analysis^55^. The MMC was moved and rotated in the simulation system by the treatment team until the ideal position was achieved and approved. The dental midline (translation) and occlusal plane (roll rotation) were first modified in the frontal view. Subsequently, in the lateral view, the occlusal plane (pitch rotation) and facial profile were adjusted. The basal view of the 3D image was referred to for verifying the symmetry and collision between the ramus segments and the mandibular body contour (yaw rotation). These processes were repeated and were finally used as the virtual 3D planning model (refer to the video). The new position was labeled P2. Each 3D surgical simulation was performed twice by two operators and measurement in P2 was used to investigate the intraobserver reproducibility and interobserver reliability.

In summary, the surgical plan from conventional 2D cephalometry was transferred to the 3D imaging system, producing the P1 model. The P1 images were evaluated and modified by moving the maxillomandibular complex if it was found to have problems in midline, proportion, bony collision, etc. The final maxillomandibular position was named P2.

### Comparison of the P1 and P2 models

To compare the differences in P1 and P2, image registration and superimposition of the cranial base were performed in the two models by using the best-fit method. The accuracy of the cranial base registration was verified by observing the distance color map between the registered P1 and P2 images. The deviation value was automatically calculated, and the value of 0.5 mm or less was considered acceptable to ensure that the corresponding reference areas had the maximum possible accuracy^52^,^53^. After the registration, the 23 reference landmarks could be located separately in P1 and P2 model for differences comparison. The two models used the same coordinate system to measure changes in hard tissue landmarks.

**Figure 6:**
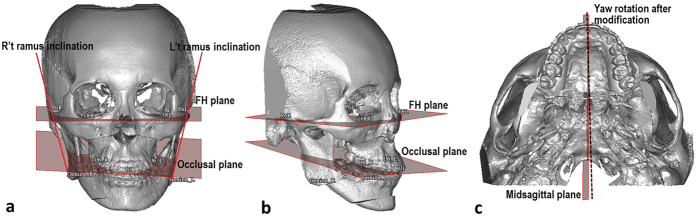
Angular measurement of yaw, roll, pitch rotation and ramus inclination on 3D model. Frontal view (**a**), Oblique view (**b**) and Basal view (**c**) (dashed line shows the yaw rotation after modification). Pitch rotation is the angle between lateral occlusal plane to FH plane. Roll rotation is the angle formed by FH plane and bimaxillary first molar line. Yaw rotation is the angle between sagittal plane and midpalatal suture. Frontal ramus inclination is the angle between FH plane and line from lateral condyle point to lateral gonion point.

Twenty-three landmarks and 3 reference planes were defined on each 3D model to conduct 14 linear and 4 angular measurements (Table 4 and Fig. 6). Changes of the landmarks in the FH plane, coronal plane, and sagittal plane were calculated. The 3 planes are defined in Table 4. Angular measurements, namely yaw, roll, and pitch rotation and bilateral ramus inclination in the frontal view, were performed from P1 to P2. Six parameters—midline, genioplasty, pitch, yaw, roll and ramus inclination—were used to evaluate the positional changes in the MMC. Changes in these parameters revealed the revisions of the 2D surgical plan (P1) after the 3D simulation (P2). Ten randomly selected cases were used for error assessment. The landmarks were located and digitized in each 3D model. The same examiner repeated the procedure 1 month later, and displacements of the landmarks were measured.

### Surgical implementation

After 3D simulation, the surgery was executed according to the 3D planning by the senior surgeon (LJL). Two-jaw orthognathic surgery by using a single occlusal splint was applied^56^,^57^. The maxilla and mandible were mobilized after implementing LeFort I and bilateral sagittal split osteotomy^58^,^59^. The maxillary and mandibular segments were placed in the intermaxillary fixation along with the final occlusal splint. The MMC was repositioned using the positioning guides, and the maxilla was fixed on each nasomaxillary and zygomaticomaxillary buttress. The MMC positioning guides were designed using CAD software (Geomagic Wrap software, 3D System, USA) and fabricated using a 3D printer (Objet30 OrthoDesk jets, Stratasys Ltd. Nasdaq: SSYS) according to the 3D plan of the maxillary position^60^. Because a single occlusal splint method was used, the MMC location was determined by positioning the maxilla. The positioning guides were matching to the planed movement of the LeFort I segment and creating a hook-shaped wedge in the piriform aperture for stabilization. The hook-shaped design allowed fast and accurate positioning of the maxilla during surgery. An adequate contact area was required, superiorly from infraorbital foramen and inferiorly to gingiva. Drill holes were created for temporary fixation of the guides and the maxilla^60^.

### Postoperative validation

CBCT images were captured 1 month after surgery. To evaluate the accuracy of the virtual surgical planning with respect to the actual surgery, the surgical simulation model (P2) and the postoperative CBCT image were registered using surface registration of the cranial base and the upper facial bones. After the initial registration, the images were superimposed to quantify the difference between the images. The accuracy of the surface superimposition was calculated in terms of the root-mean-square deviation (RMSD) of distance between the superimposed model, with RMSD ≤ 0.5 mm considered acceptable (Fig. 4)^61^,^62^. Differences between the two models were computed for validation.

### Patient satisfaction

In follow-up visits at least 6 months after surgery, patients were inquired about their level of satisfaction with the dental alignment and facial appearance.

### Statistical analysis

To evaluate intraobserver reproducibility and interobserver reliability of measurements, the intraclass correlation coefficients (ICCs) of the errors as well as the means and standard deviations were calculated. A paired t-test was used to evaluate the significance of the differences in linear and angular measurement, with p < 0.05 considered significant.

## Additional Information

**How to cite this article**: Ho, C.-T. *et al*. Three-dimensional surgical simulation improves the planning for correction of facial prognathism and asymmetry: A qualitative and quantitative study. *Sci. Rep.* **7**, 40423; doi: 10.1038/srep40423 (2017).

**Publisher's note:** Springer Nature remains neutral with regard to jurisdictional claims in published maps and institutional affiliations.

## Supplementary Material

Supplementary Information

Supplementary Video

## Figures and Tables

**Table 1:** Absolute linear changes of landmarks in x, y, z axis between 2D plan and 3D plan (3D image models registered at the cranial base).

Landmark	X-axis				Y-axis				Z-axis			
	2D	3D	Difference		2D	3D	Difference		2D	3D	Difference	
	Mean(SD)	Mean(SD)	Mean(SD)	p value*	Mean(SD)	Mean(SD)	Mean(SD)	p value*	Mean(SD)	Mean(SD)	Mean(SD)	p value*
A	79.30 (3.76)	78.17 (3.98)	1.11 (1.71)	0.03	−121.61 (9.56)	−123.11 (9.54)	0.50 (0.95)	0.50	−145.35 (4.40)	−146.06 (4.53)	0.71 (1.01)	0.13
B	79.60 (3.80)	78.32 (3.51)	1.24 (1.53)	0.01	−118.03 (10.81)	−118.62 (10.49)	0.59 (0.90)	0.30	−184.77 (4.78)	−185.78 (4.69)	1.02 (1.43)	0.03
U3L	94.50 (18.22)	93.17 (18.00)	1.35 (1.85)	0.01	−114.22 (23.81)	−115.06 (23.90)	0.84 (1.09)	0.05	−161.61 (30.85)	−162.47 (31.08)	0.86 (1.17)	0.05
U3R	59.40 (12.04)	57.91 (11.74)	1.53 (2.02)	0.00	−113.27 (23.84)	−114.19 (23.89)	0.92 (1.52)	0.07	−160.40 (31.08)	−162.04 (30.93)	0.81 (1.35)	0.05
U6L	101.70 (19.56)	100.09 (19.27)	1.61 (1.81)	0.00	−96.34 (21.10)	−97.36 (21.23)	1.01 (1.15)	0.01	−157.78 (30.22)	−158.73 (30.46)	0.95 (1.32)	0.04
U6R	53.70 (4.68)	52.07 (4.49)	1.62 (1.75)	0.00	−99.79 (11.03)	−100.96 (10.94)	1.17 (1.63)	0.02	−162.84 (4.69)	−164.08 (4.75)	1.24 (1.50)	0.01
UI	79.50 (4.12)	78.23 (4.41)	1.31 (1.95)	0.02	−125.30 (9.91)	−125.95 (9.59)	0.65 (1.24)	0.26	−167.39 (4.68)	−168.17 (5.13)	0.77 (1.16)	0.10
L3L	93.70 (3.59)	92.34 (3.80)	1.31 (1.85)	0.01	−116.23(9.31)	−117.05 (9.07)	0.82 (1.09)	0.06	−166.12 (5.10)	−166.91 (5.51)	0.79 (1.17)	0.09
L3R	65.60 (4.50)	64.30 (4.49)	1.32 (1.81)	0.01	−115.70 (8.71)	−116.49 (8.40)	0.78 (1.41)	0.14	−165.95 (5.14)	−166.86 (5.59)	0.91 (1.36)	0.06
L6L	99.60 (19.25)	98.11 (18.89)	1.52 (1.62)	0.00	−94.98 (21.27)	−95.85 (21.35)	0.87 (1.04)	0.03	−158.69 (30.34)	−159.59 (30.56)	0.90 (1.34)	0.06
L6R	54.60 (10.95)	53.10 (10.54)	1.52 ((1.61)	0.00	−94.43 (20.69)	−95.32 (20.84)	0.89 (1.25)	0.04	−158.77 (30.32)	−159.65 (30.51)	0.89 (1.25)	0.05
LI	79.90 (4.06)	78.65 (3.76)	1.22 (1.66)	0.01	−123.08 (9.55)	−123.60 (9.28)	0.52 (0.96)	0.45	−167.97 (4.90)	−168.61 (5.22)	0.64 (1.01)	0.23
Pog	79.70 (3.70)	78.38 (3.32)	1.33 (1.64)	0.00	−118.15 (10.33)	−118.99 (9.92)	0.84(0.99)	0.03	−202.68 (4.48)	−203.33 (4.68)	0.65 (1.07)	0.22
Me	79.70 (3.61)	78.33 (3.24)	1.36 (1.68)	0.00	−115.09 (10.55)	−116.04 (10.22)	0.95 (1.09)	0.02	−206.04 (4.63)	−206.71 (4.77)	0.66 (1.10)	0.21

^*^The difference was considered statistically significant if p < 0.05.

**Table 2:** Absolute angular change of the maxillomandibular complex between P1 model (2D plan) and P2 model (3D plan).

Parameters	2D planMean (SD)	3D planMean (SD)	DifferenceMean (SD)	p value*
Pitch	13.50 (4.04)	15.23 (4.03)	1.73 (0.41)	0.00
Roll	1.43 (1.17)	0.37 (0.41)	1.06 (0.23)	0.01
Yaw	0.00 (0.00)	1.88 (1.73)	1.88 (0.32)	0.00
Difference of frontal ramus inclination	4.15 (3.06)	0.78 (1.47)	3.37 (3.21)	0.00

^*^The difference was considered statistically significant if p < 0.05.

**Table 3:** The absolute root-mean-square distance (RMSD) for the maxilla, mandible and maxillomandibular complex comparing post-surgical 3D image and 3D simulation model.

	Forehead+ Orbital	Maxilla+ Mandible	Maxilla	Mandible
**Mean**	0.43	0.77	0.63	0.85
**SD**	0.02	0.36	0.25	0.41

The forehead and orbital areas were used for registration of the two images. The data were in mm.

**Table 4:** Definitions of 3D cephalometric landmarks and reference planes.

Landmark	Abbreviation	Definition
Orbitale	OrLOrR	The most inferior point of left infraorbital rimThe most inferior point of right infraorbital rim
Porion	PoL,PoR	The highest points of the left external acoustic meatusThe highest points of the right external acoustic meatus
Average orbitale	OrA	Midpoint between the OrR and OrL.
Nasion	N	The middle point of the frontonasal suture
Anterior nasal spine	ANS	The most anterior midpoint of the anterior nasal spine of the maxilla
Point A	A	The innermost point on the contour of the premaxilla between ANS and U1
Point B	B	The innermost point on the contour of the mandible between bony chin and L1
Upper incisor	U1	Midoint between the crowns of the maxillary central incisors
Lower incisor	L1	Midpoint contact between the crowns of the mandibular central incisors.
Pogonion	Pog	The most anterior midpoint of the chin on the outline of the mandibular symphysis
Menton	Me	The most inferior midpoint of the chin on the outline of the mandibular symphysis.
Maxillary canine	U3LU3R	Cusp of the left maxillary canineCusp of the right maxillary canine
Mandibular canine	L3LL3R	Cusp tip of the left mandibular canineCusp tip of the right mandibular canine
First maxillary molar	U6LU6R	Mesio-buccal cusp of the left first maxillary molarMesio-buccal cusp of the right first maxillary molar
Lateral condyle point	Cd latLCd latR	The most lateral point of left condyle headThe most lateral point of right condyle head
Lateral gonion	Go latLGo latR	The most lateral point of left gonionThe most lateral point of right gonion
Frankfort horizontal plane	FH plane	A plane passing PoL, PoR, and OrA
Coronal plane		A plane perpendicular to FH plane, through right and left porion
Sagittal plane		A plane perpendicular to the FH and coronal planes, through nasion
