# Supplementary material for: Three-dimensional surgical simulation improves the planning for correction of facial prognathism and asymmetry: A qualitative and quantitative study
Source: Sci Rep. 2017 Jan 10;7:40423. doi: 10.1038/srep40423 (PMC5223192; doi:10.1038/srep40423)
Supplement: Supplementary Information [file srep40423-s1.pdf]

Three-dimensional surgical simulation improves the planning for correction of facial prognathism and asymmetry: A qualitative and quantitative study

Cheng-Ting Ho[1], Hsiu-Hsia Lin[2], Eric JW Liou[1], Lun-Jou Lo[3,*]

[1]Department of Craniofacial Orthodontics, Department of Dentistry, Chang Gung Memorial Hospital, Taoyuan, Taiwan

[2]Department of Craniofacial Research Center, Chang Gung Memorial Hospital, Taoyuan, Taiwan

[3]Department of Plastic & Reconstructive Surgery, and Craniofacial Research Center, Chang Gung Memorial Hospital, Chang Gung University, Taoyuan, Taiwan

*** Corresponding Author**

Lun-Jou Lo, M.D. Department of Plastic & Reconstructive Surgery, Chang Gung Memorial Hospital. 5, Fu-Shin Street, Kwei Shan, Taoyuan, Taiwan 333. Tel: 886-3-3281200, ext 2430. Fax: 886-3-3271029. Email: lunjoulo@cgmh.org.tw

Video Legends

Time	Video legends
00:00~00:05	This gentleman complained prognathism, chin deviation to right side, and long face.
00:05~00:10	Initial treatment plan was made using traditional 2D cephalometric study.
00:10~00:18	The 2D surgical plan was transferred to the 3D simulation system and an initial virtual surgical model was created.
00:20~00:32	The distal mandibular segment was first moved to occlude with the maxilla using the final occlusal splint, forming the maxillomandibular complex (so called the MMC).
00:34~00:40	The MMC was mobilized to the position according to the 2D surgical plan.
00:41~00:47	If the MMC position was not satisfied, then a modification would be made.
00:49~00:57	Modifications of the MMC position was first performed to the dental midline and occlusal plane in the frontal view.
00:59~01:04	Then in the lateral view, adjusted the occlusal plane cant and the facial bony profile.
01:05~01:10	After following occlusion, then the 3D simulation was performed.
01:12~01:15	We rotated the MMC to meet the best fit.
01:18~01:27	Look at the other side, after following occlusion, the 3D simulation was performed. We rotated the MMC to meet the best fit.
01:31~01:41	In the basal view, we check the collision between ramus segments, the symmetry from both sides, and the mandibular body contour. Yaw rotation was performed, and the ramus proximal segments were adjusted.
01:47~01:51	Some measurements were performed from different views according to the 3D planning.
01:53~01:59	These views and the measurement data provided important surgical information.
02:03~02:10	Constructed images for the inferior alveolar nerve were given to prevent nerve injury during ramus splitting.
02:12~02:15	The patient obtained satisfactory results.
